# Post-COVID-19 syndrome among hospitalized COVID-19 patients: a cohort study assessing patients 6 and 12 months after hospital discharge

**DOI:** 10.1590/0102-311XEN027423

**Published:** 2024-02-19

**Authors:** Roseany Patricia Silva Rocha, Amanda Cristina de Souza Andrade, Francine Nesello Melanda, Ana Paula Muraro

**Affiliations:** 1 Universidade do Estado de Mato Grosso, Diamantino, Brasil.; 2 Instituto de Saúde Coletiva, Universidade Federal de Mato Grosso, Cuiabá, Brasil.; 3 Universidade Estadual de Londrina, Londrina, Brasil.

**Keywords:** COVID-19, Post-Acute COVID-19 Syndrome, Hospitals, COVID-19, Síndrome Pós-COVID-19 Aguda, Hospitais, COVID-19, Síndrome Post Agudo de COVID-19, Hospitales

## Abstract

Post-COVID-19 syndrome involves a variety of symptoms that last more than 12 weeks after COVID diagnosis. This study aimed to analyze post-COVID-19 syndrome among hospitalized COVID-19 patients 6 and 12 months after hospital discharge. This is an ambidirectional cohort study conducted with individuals who were discharged from three main hospitals in the capital of Mato Grosso State, Brazil, between October and December 2021 and January and March 2022. After data collection from medical records, the individuals were interviewed by telephone 6 and 12 months after hospital discharge, when they were asked about the presence of ongoing or new symptoms and when symptom frequency was evaluated according to sociodemographic and economic characteristics hospitalization, and health conditions. Of all 277 medical records evaluated, 259 patients were eligible to participate in the study, 190 patients six months after discharge and 160 patients 12 months after hospital discharge. At six months, 59% were female patients, 40% were aged 60 years or older, and 87.4% reported at least one symptom. At 12 months, 58.7% were female patients, 37.5% were aged 30 to 49 years, and 67.5% reported at least one symptom. Fatigue was the most common symptom 6 and 12 months after hospital discharge (55.3% and 40.6%, respectively), followed by memory problems (36.8%; 20%), and hair loss (26.8%; 11.2%). The prevalence of post-COVID-19 syndrome was higher among patients of older age, lower income, with hypertension, diabetes, and more severe infection during hospitalization. The risk factors for post-COVID-19 syndrome help understand the long-term effects and the importance of monitoring after the acute phase of the disease.

## Abstract (pt)

A síndrome pós-COVID-19 é um termo usado para descrever um conjunto diversificado de sintomas que persistem por mais de 12 semanas da infecção diagnosticada. O objetivo deste estudo foi analisar a síndrome pós-COVID-19 entre hospitalizados por COVID-19 após 6 e 12 meses da alta hospitalar. Trata-se de estudo de coorte ambidirecional, realizado com indivíduos que receberam alta em três dos principais hospitais da capital de Mato Grosso, Brasil, entre outubro e dezembro de 2021 e janeiro e março de 2022. Após coleta de dados em prontuários, os indivíduos foram entrevistados por telefone após 6 e 12 meses da alta hospitalar, sendo questionados sobre a presença de sintomas persistentes ou novos, para a avaliação de sua frequência segundo características sociodemográficas, econômicas, relativas à internação e condições de saúde. Dos 277 prontuários avaliados, 259 pacientes foram elegíveis para o estudo, 190 aos seis meses e 160 após 12 meses da alta hospitalar. Aos seis meses, 59% eram mulheres, 40% com 60 anos ou mais de idade e 87,4% referiram a presença de pelo menos um sintoma. Aos 12 meses, 58,7% eram mulheres, 37,5% com 30 a 49 anos e 67,5% referiram a presença de pelo menos um sintoma. A fadiga foi o sintoma mais comum após 6 e 12 meses de alta hospitalar (55,3% e 40,6%, respectivamente), seguido de problemas de memória (36,8%; 20%) e perda de cabelo (26,8%; 11,2%). Foi maior a prevalência de síndrome pós-COVID-19 entre indivíduos de maior faixa etária, menor renda, hipertensos, diabéticos e com maior gravidade durante a internação. Os fatores de risco da síndrome pós-COVID-19 contribuem para a compreensão dos efeitos a longo prazo e da importância do acompanhamento após a fase aguda da doença.

## Abstract (es)

El síndrome post-COVID-19 es un término utilizado para describir un conjunto diversificado de síntomas que persisten durante más de 12 semanas de la infección diagnosticada. El objetivo fue analizar el síndrome post-COVID-19 entre hospitalizados por COVID-19 tras 6 y 12 meses del alta hospitalaria. Se trata de un estudio de cohorte ambidireccional, realizado con individuos que fueron dados de alta en tres de los principales hospitales de la capital de Mato Grosso, Brasil, entre octubre y diciembre de 2021 y enero y marzo de 2022. Tras recolectar los datos en registros médicos, se entrevistaron los individuos por teléfono tras 6 y 12 meses del alta hospitalaria, cuestionándoles sobre la presencia de síntomas persistentes o nuevos y evaluando su frecuencia conforme las características sociodemográficas, económicas, relacionadas con la hospitalización y condiciones de salud. De los 277 registros médicos evaluados, se eligieron 259 pacientes para el estudio, 190 a los 6 meses y 160 tras 12 meses del alta hospitalaria. A los 6 meses, el 59% eran mujeres, el 40% tenían 60 años o más y el 87,4% refirieron la presencia de al menos un síntoma. A los 12 meses, el 58,7% eran mujeres, el 37,5% tenían entre 30 y 49 años y el 67,5% refirieron la presencia de al menos un síntoma. La fatiga fue el síntoma más común tras 6 y 12 meses del alta hospitalaria (el 55,3% y el 40,6%, respectivamente), seguido de los problemas de memoria (el 36,8% y el 20%) y caída del pelo (el 26,8% y el 11,2%). La prevalencia de síndrome post-COVID-19 fue más alta entre los individuos de mayor edad, menor renta, hipertensos, diabéticos y con mayor gravedad durante la hospitalización. Los factores de riesgo del síndrome post-COVID-19 contribuyen para la comprensión de los efectos a largo plazo y de la importancia del seguimiento tras la fase aguda de la enfermedad.

## Introduction

The COVID-19 pandemic, caused by the novel coronavirus (SARS-CoV-2), is a public health emergency of global impact ^1^. By January 2023, it had affected more than 600 million people worldwide ^2^; more than 35 million in Brazil, representing a cumulative incidence of 17.35 per 10,000 inhabitants ^3^. Specifically in the state of Mato Grosso, this number has exceeded 838,000 contaminated people, with 15,246 deaths due to the disease ^4^. With the progression of the COVID-19 pandemic, there is a growing awareness of its long-term impacts on the population health ^5^. Currently, SARS-CoV-2 is recognized as a condition that causes not only pulmonary issues, but also a multiple organ syndrome ^6^.

After the initial acute infection, like many other viral disorders, a variety of long-lasting and new symptoms of COVID-19 has been described using the following terminologies: post-COVID-19 syndrome, long COVID, and post-COVID-19 conditions ^7^. These terminologies refer to symptoms that develop during or after a SARS-CoV-2 infection and that last more than 12 weeks after the infection is diagnosed ^8^ ^,^ ^9^ ^,^ ^10^.

According to estimates, persistent COVID-19 symptoms are observed in about one out of ten cases of the disease ^10^; however, in Brazil, a study conducted in São Paulo showed that 68% of hospitalized patients had at least one recurrent symptom related to COVID-19 11 months after diagnosis ^11^.

The frequency of persistent symptoms depends on the extension and severity of the infection and organs affected ^12^. According to a systematic review of longitudinal studies, the most frequently reported symptoms were fatigue, dyspnea, memory loss, and being unable to concentrate up to one year after recovery from COVID-19 ^13^. The same review also showed that female patients, older age, comorbidities, and disease severity in the acute phase of the disease are risk factors for developing post-COVID-19 syndrome ^13^.

Also, post-COVID-19 syndrome should be evaluated in relation to the dominant variant of the SARS-CoV-2. Since the first case, several variants have been identified, with new waves and mortality caused by the disease ^14^. COVID-19 vaccines also follow the pace of variant identification for their improvement ^15^. In late 2021, the Omicron variant (PANGO B.1.1.529) caused a sudden increase in cases worldwide, but according to a recent study, the chance of post-COVID-19 syndrome was lower among cases due to Omicron variant when compared to the Delta variant (the one identified before Omicron) ^16^.

In this dynamic context of the COVID-19 pandemic, post-COVID-19 syndrome should be characterized over time, as well as the factors associated with this condition. In addition to many patients requiring specialized monitoring, a number of patients need to be readmitted to hospitals ^10^, which means an increase in healthcare treatments ^3^. These results agree with findings of a systematic review ^10^, which also highlighted that a considerable number of participants reported losses in professional, social/family, and mental health areas.

Considering the above, this study aimed to analyze post-COVID-19 syndrome among hospitalized COVID-19 patients 6 and 12 months after hospital discharge.

## Methods

This is an ambidirectional cohort study that assessed patients with a diagnosis of COVID-19 living in Cuiabá (capital of Mato Grosso) and Várzea Grande (a municipality near the capital), and who were admitted to public and private hospitals in Cuiabá, with cases ending up with hospital discharge between October and December 2021 and January and March 2022. The cases were identified by IndicaSUS, a system implemented by the government of Mato Grosso ^4^, accessed through the Cuiabá Municipal Health Department. After collecting data from medical records, individuals were interviewed by telephone 6 and 12 months after hospital discharge.

Of all seven hospitals in Cuiabá, only three allowed access to patient records. Individuals aged 18 or over, with COVID-19 confirmed through PCR, rapid test or antigen test, and who were discharged from hospital according to information recorded in IndicaSUS were considered eligible. Exclusion criteria were patients living in a nursing home and patients who have communication difficulties (aphasia, cognitive impairment, and severe hearing loss).

**Figure 1:**
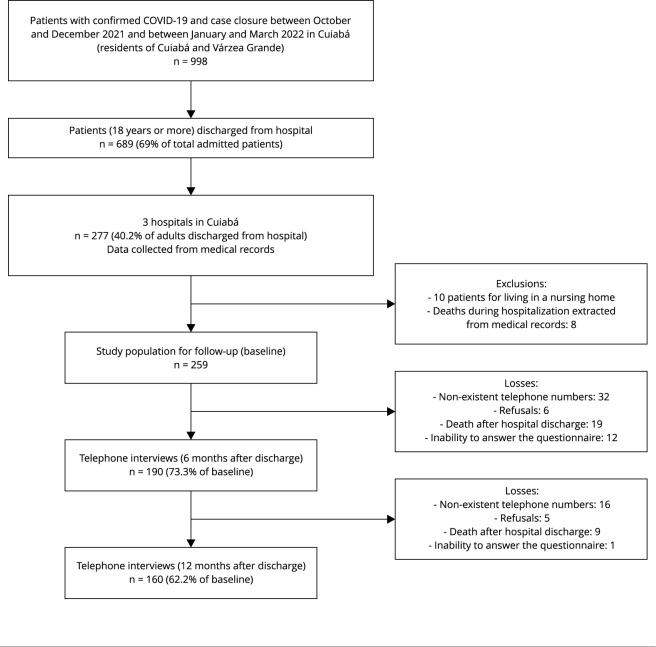
Flowchart show the selection of patients who were admitted and interviewed six months after hospital discharge. Cuiabá, Mato Grosso State, Brazil, 2022.

From October 2021 to March 2022, 998 patients with confirmed COVID-19 living in Cuiabá and Várzea Grande had their cases closed in hospitals in the capital; of these, 689 were discharged (69%), with 277 in the three hospitals selected for the study (40.2% of the total adult patients who were discharged during the period). During data collection from medical records, 10 patients were excluded because they lived in nursing homes and 8 were excluded because, despite being registered in IndicaSUS as hospital discharges, they died during hospitalization. Therefore, 259 individuals remained eligible for the study (Figure 1).

Participants were contacted via telephone and messaging application and invited to participate in the study, scheduled according to the interviewee’s preferences. During data collection, the study objective, informed consent form, free decision to participate, privacy guarantee, and the possibility of withdrawal from the study were explained to participants.

To identify deaths after hospital discharge, a search was conducted in the Brazilian Mortality Information System for individuals not found through telephone contact 12 months after hospital discharge. The search was performed by technicians from the Cuiabá Municipal Health Department using personal information from medical records (name, taxpayer identification number, birth date, and mother’s name). In addition to these, patients who refused to participate and who reported impossibility to be contacted by telephone and inability to answer the questionnaire due to communication difficulties such as aphasia, cognitive impairment or severe hearing loss, in addition to deaths after hospital discharge, were considered losses.

Individual telephone interviews were conducted with participants and lasted about 20 to 35 minutes. The questions referred to demographic and socioeconomic characteristics, housing and living conditions, health and hospitalization characteristics, and persistent symptoms since the acute phase of COVID-19 or new symptoms at the time of the interview.

The variables about sociodemographic and economic characteristics were: sex, age group in years (18-29, 30-49, 50-59, and 60 or more), ethnicity/skin color (white, mixed-race, black, yellow/Indigenous), education (completed elementary school, completed high school, completed higher education or more), monthly income in minimum wages (up to 2, 2-3, and 3 or more), whether the patient had a job at the time of hospital admission and at the time of the interview (among those who reported a job when they were admitted).

The variables about health and hospitalization characteristics were: diagnosis of COVID-19 prior to the infection that caused hospitalization, number of vaccine doses received before hospitalization (1, 2, 3, or 4), self-reported comorbidities (high blood pressure, diabetes mellitus, obesity, heart disease), number of comorbidities (no comorbidity, 1 or 2, and 3 or more). The variables obtained from medical records were: length of stay (average days classified into tertiles), intensive care unit (ICU) hospitalization (yes/no), days in the ICU (average days classified into tertiles), and mechanical ventilation required (yes/no).

The following comorbidities: high blood pressure, diabetes mellitus, obesity, and heart disease were considered in the data analysis, as they were the most frequent among reported comorbidities. However, only 11 patients reported obesity. When body mass index (BMI) was calculated using weight and height data, 46 individuals presented 30kg/m^2^ or more, characterizing obesity ^17^, with this information considered in data analysis.

Participants were also asked about 24 classes of symptoms present in the acute phase of COVID-19 and 6 and 12 months after hospital discharge - this list of symptoms was developed according to the literature ^18^. A blank field was provided for the patient to report symptoms that were not mentioned during the interview.

Symptoms were classified as: muscular (tiredness/fatigue, joint pain, asthenia/muscle weakness, dysphoria/indisposition); neuropsychiatric (memory problems, anxiety, attention deficit/concentration problems, dizziness, depression, headache, taste and smell disorders, sleep disorders, low mood, post-traumatic stress disorder); dermatological (hair loss), cardiovascular (palpitation, dyspnea, chest pain); and pulmonary (dyspnea, cough, mechanical ventilation required). Other symptoms were also investigated: sore throat, runny nose, fever, sweating, nausea ^18^. Also, the ten most frequent symptoms in the acute phase of the disease were evaluated regarding the presence of persistent symptoms six months after hospital discharge. Post-COVID-19 syndrome was defined as the presence of persistent or new symptoms 6 and 12 months after hospital discharge, as reported in previous studies ^5^ ^,^ ^10^ ^,^ ^19^. The number of symptoms (none, 1 or 2, 3 or more) was also evaluated.

The analyses were performed in Stata, version 16 (https://www.stata.com). Variables were described using absolute and relative frequency, with the chi-square test or Fisher’s exact test applied to assess differences between proportions, considering the statistical significance level of 5% (p-value < 0.05).

All ethical aspects in research were respected, in compliance with *Resolution n. 466/2012* of the Brazilian National Health Council. This study was approved by the Research Ethics Committee with Human Beings in the Health Area of the Mato Grosso Federal University, Cuiabá Campus (report n. 5,415,255/2022 of May 18, 2022). All participants signed the informed consent form.

## Results

In total, 190 patients were evaluated six months after hospital discharge (mean: 6.29 months, standard deviation - SD: 1.24) and 160 patients 12 months after hospital discharge (mean: 13.17 months, SD: 1.11), representing 73.3% and 62.2% of the eligible population, respectively. Among the losses and exclusions at 6 and 12 months, the highest proportions referred to impossibility to be contacted by telephone (n = 48), refusal to participate (n = 11), inability to answer the questionnaire due to communication difficulties such as of aphasia, cognitive impairment or severe hearing loss (n = 13), in addition to deaths after hospital discharge (n = 28) (Figure 1).

Of all 190 individuals assessed six months after hospital discharge, 59% were female patients, 40% were 60 years old or over, 63.2% reported mixed-race skin, 55.8% had completed elementary education, and 57.4% had a job when they were hospitalized but only 41.6% had a job when the interview was conducted. When asked about the presence of symptoms, 87.4% reported COVID-19 symptoms at the time of the interview, conducted six months after hospital discharge, and 83.2% did not think they were recovered from COVID-19. At the evaluation of 24 symptoms, 88.4% reported at least one symptom and 36.3% had three or more symptoms (Table 1).

**Table 1:** Post-COVID-19 syndrome * and number of symptoms among patients assessed 6 and 12 months after hospital discharge, according to sociodemographic and economic characteristics. Cuiabá, Mato Grosso State, Brazil, 2022.

Characteristics	6 months				12 months			
	Total (n = 190)	Post-COVID-19 syndrome	Symptoms		Total (n = 160)	Post-COVID-19 syndrome	Symptoms	
		Yes	1 or 2	3 or more		Yes	1 or 2	3 or more
	n (%)	n (%)	n (%)	n (%)	n (%)	n (%)	n (%)	n (%)
General	190 (100.0)	168 (88.4)	99 (52.1)	69 (36.3)	160 (100.0)	110 (68.7)	97 (60.6)	13 (8.1)
Sex								
Male	78 (41.0)	69 (88.5)	47 (60.3)	22 (28.2)	66 (41.3)	46 (70.7)	44 (67.7)	2 (3.1)
Female	112 (59.0)	99 (88.4)	52 (46.5)	47 (41.9)	94 (58.7)	64 (67.4)	53 (55.8)	11 (11.6)
p-value **	0.99		0.13		0.36		< 0.01	
Age group (years)								
18-29	23 (12.1)	15 (65.2)	11 (47.8)	4 (17.4)	20 (12.5)	8 (40.0)	8 (40.0)	-
30-49	62 (32.6)	50 (80.6)	35 (56.4)	15 (24.2)	60 (37.5)	40 (66.7)	34 (56.7)	6 (10.0)
50-59	29 (15.3)	28 (96.5)	16 (55.2)	12 (41.4)	29 (18.1)	26 (89.6)	21 (72.4)	5 (17.2)
60 or more	76 (40.0)	75 (98.7)	37 (48.7)	38 (50.0)	51 (31.9)	36 (70.6)	34 (66.7)	2 (3.9)
p-value **	< 0.01		< 0.01		< 0.01		< 0.01	
Ethnicity/Skin color								
White	36 (18.9)	27 (75.0)	26 (54.2)	13 (27.1)	34 (21.2)	18 (52.9)	17 (50.0)	1 (2.9)
Mixed-race	120 (63.2)	108 (90.0)	59 (50.4)	47 (40.2)	96 (60.0)	75 (78.1)	68 (70.8)	7 (7.3)
Brack	8 (4.2)	8 (100.0)	10 (52.6)	7 (36.9)	7 (4.4)	5 (71.4)	5 (71.4)	-
Yellow/Indigenous	11 (5.8)	10 (90.9)	4 (66.7)	2 (33.3)	10 (6.2)	7 (70.0)	3 (30.0)	4 (40.0)
Ignored	15 (7.9)	15 (100.0)			13 (8.2)	5 (38.4)	4 (30.7)	1 (7.7)
p-value **	0.29		0.50		< 0.01		0.42	
Education								
Completed elementary	106 (55.8)	97 (91.5)	50 (47.2)	47 (44.3)	80 (50.0)	58 (72.5)	49 (61.2)	9 (11.2)
Completed high school	51 (26.8)	42 (82.5)	29 (56.9)	13 (25.5)	49 (30.6)	34 (69.4)	30 (61.2)	4 (8.1)
Completed Higher education or more	33 (17.4)	29 (87.9)	20 (60.6)	9 (27.3)	31 (19.4)	18 (58.1)	18 (58.1)	-
p-value **	0.24		0.09		0.33		0.28	
Monthly income (minimum wages)								
Up to 2	59 (32.1)	58 (98.3)	29 (49.1)	29 (49.2)	48 (31.2)	41 (85.4)	35 (72.9)	6 (12.5)
2-3	59 (32.1)	52 (88.1)	36 (61.1)	16 (27.1)	46 (29.9)	33 (71.7)	29 (63.0)	4 (8.7)
3 or more	66 (35.8)	55 (83.3)	33 (50.0)	22 (33.3)	60 (39.0)	33 (55.0)	30 (50.0)	3 (5.0)
p-value **	0.02		< 0.01		< 0.01		< 0.01	
Job at hospital admission								
Yes	109 (57.4)	95 (87.2)	62 (56.9)	33 (30.3)	99 (61.8)	74 (74.7)	62 (62.6)	12 (12.1)
No	81 (42.6)	73 (90.1)	37 (45.7)	36 (44.4)	81 (38.2)	36 (59.0)	35 (57.4)	1 (1.6)
p-value **	0.53		0.13		0.03		< 0.01	
Job after hospital discharge								
Yes	79 (41.6)	63 (79.8)	45 (60.0)	18 (22.8)	86 (53.7)	55 (63.9)	51 (59.3)	4 (4.6)
No	111 (58.4)	105 (94.6)	54 (48.6)	51 (46.0)	74 (46.2)	55 (74.3)	46 (62.1)	9 (12.1)
p-value **	< 0.01		< 0.01		0.15		0.12	

* Presence of one or more symptoms related to COVID-19 six months after hospital discharge;

** Chi-square test or Fisher’s exact test.

At the 12-month interview, 160 individuals were evaluated. Of these, 58.7 were female patients, with similar sociodemographic characteristics to the 6-month interview. When asked about the symptoms, 67.5% reported COVID-19 symptoms at the time of the interview, conducted 12 months after hospital discharge, and 66.8% did not think they were recovered from COVID-19. At the evaluation of 24 symptoms, 68.7% reported at least one symptom and 8.13% had three or more symptoms (Table 1).

No significant difference was found in the presence of post-COVID-19 syndrome according to sex at the 6-month assessment; however, at the 12-month assessment, the prevalence of three or more symptoms was higher among female patients (11.6%) when compared to male patients (3.1%). No significant difference was observed in the following variables: ethnicity/skin color, education, and employment at the time of hospitalization. The frequency of post-COVID-19 syndrome was higher among individuals of older age, mixed-race skin color, and lower family income, and among those who had no job when the interview was conducted 6 and 12 months after hospital discharge. When evaluated after 12 months, the prevalence of persistent symptoms did not present a statistically significant difference. The presence of three or more symptoms was significantly higher among patients who reported no job (46%) when compared to those who had a job (22.8%) (Table 1).

Only 9.4% of individuals had already been diagnosed with COVID-19 prior to the infection that caused hospitalization, with no significant difference with the presence of post-COVID-19 syndrome 6 and 12 months after hospital discharge. However, among patients who were hospitalized due to recurrent COVID-19 infection, 64.7% had three or more symptoms after six months versus 34.4% of patients who had been hospitalized due to the first infection, with a borderline significance level (p-value = 0.05); but this difference was not observed 12 months after discharge. More than 70% of interviewers had received at least two doses of the vaccine against SARs-CoV-2 before hospitalization (41.6% received two doses and 32.6% received 3 or 4 doses), with no significant difference in relation to post-COVID-19 syndrome after 6 and 12 months (Table 2).

**Table 2:** Post-COVID-19 syndrome * and number of symptoms among patients assessed 6 and 12 months after hospital discharge, according to hospitalization characteristics. Cuiabá, Mato Grosso State, Brazil, 2022.

Characteristics	6 months				12 months			
	Total (n = 190)	Post-COVID-19 syndrome	Symptoms		Total (n = 160)	Post-COVID-19 syndrome	Symptoms	
		Yes	1 or 2	3 or more		Yes	1 or 2	3 or more
	n (%)	n (%)	n (%)	n (%)	n (%)	n (%)	n (%)	n (%)
General	190 (100.0)	168 (88.4)	99 (52.1)	69 (36.3)	160 (100.0)	110 (68.7)	97 (60.6)	13 (8.1)
COVID-19 diagnosis before infection leading to hospitalization								
No	163 (90.6)	147 (90.2)	91 (55.8)	56 (34.4)	136 (83.4)	96 (70.6)	86 (63.2)	10 (7.3)
Yes	17 (9.4)	16 (94.1)	5 (29.4)	11 (64.7)	27 (16.6)	10 (66.6)	8 (53.3)	2 (13.3)
p-value **	0.60		0.05		0.75		0.64	
Immunization at admission (dosis)								
1	32 (16.8)	25 (78.1)	16 (50.0)	9 (28.1)	30 (18.7)	19 (63.3)	19 (63.3)	-
2	79 (41.6)	71 (89.9)	43 (54.4)	28 (35.5)	68 (42.5)	48 (70.6)	39 (57.3)	9 (13.2)
3 or 4	62 (32.6)	57 (91.9)	33 (53.2)	24 (38.7)	48 (30.0)	33 (68.7)	31 (64.6)	2 (4.2)
Not vaccinated/No intention	17 (8.9)	15 (88.2)	7 (41.2)	8 (47.0)	14 (8.7)	10 (71.4)	8 (57.1)	2 (14.3)
p-value **	0.24		0.48		0.90		0.33	
Comorbidities ***								
High blood pressure								
No	79 (41.6)	59 (74.7)	42 (53.1)	17 (21.5)	73 (45.6)	39 (53.4)	34 (46.6)	5 (6.8)
Yes	111 (58.4)	109 (98.2)	57 (51.3)	52 (46.9)	87 (54.4)	71 (81.6)	63 (72.4)	8 (9.2)
p-value **	< 0.01		< 0.01		< 0.01		< 0.01	
Diabetes mellitus								
No	126 (66.3)	105 (83.3)	70 (55.5)	35 (27.8)	112 (70.0)	73 (65.2)	66 (58.9)	7 (6.2)
Yes	64 (33.7)	63 (98.4)	29 (45.3)	34 (53.1)	48 (30.0)	37 (77.1)	31 (64.6)	6 (12.5)
p-value **	< 0.01		< 0.01		0.13		0.19	
Obesity ^#^								
No	142 (75.5)	126 (88.7)	72 (50.7)	54 (38.0)	142 (88.7)	102 (67.5)	91 (60.2)	11 (7.3)
Yes	46 (24.5)	41 (89.1)	27 (58.7)	14 (30.4)	9 (5.6)	8 (88.9)	6 (66.7)	2 (22.2)
p-value **	0.94		0.613		0.18		0.16	
Heart disease								
No	182 (95.8)	163 (88.1)	98 (53.8)	62 (34.1)	152 (95.0)	105 (69.1)	94 (61.8)	11 (7.2)
Yes	8 (4.2)	5 (100.0)	1 (12.5)	7 (87.5)	8 (5.0)	5 (62.5)	3 (37.5)	2 (25.0)
p-value **	0.29		< 0.01		0.69		0.15	
Comorbidities								
None	53 (27.9)	35 (66.0)	30 (56.6)	5 (9.4)	65 (40.6)	38 (34.5)	23 (46.0)	1 (2.0)
1 or 2	125 (65.8)	121 (96.8)	66 (52.8)	55 (44.0)	46 (28.8)	32 (29.1)	67 (67.7)	11 (11.1)
3 or mais	12 (6.3)	12 (100.0)	3 (25.0)	9 (75.0)	49 (30.6)	40 (36.4)	7 (63.6)	1 (9.1)
p-value **	< 0.01		< 0.01		0.24		< 0.01	
Length of stay (days)								
Mean (SD)	12.7 (16.7)	13.6 (17.5)	14.7 (12.0)	15.9 (16.7)	12.5 (15.2)	13.6 (15.7)	13.1 (15.7)	17.1 (15.0)
p-value **	0.02		0.03		0.01		0.01	
Length of stay (tertiles)								
1st (1-6 days)	74 (39.1)	57 (77.0)	37 (50.0)	20 (27.0)	64 (86.5)	32 (50.0)	20 (57.1)	2 (6.2)
2nd (7-11 days)	56 (29.6)	53 (94.6)	28 (50.0)	25 (44.6)	49 (80.3)	41 (83.7)	16 (39.0)	5 (12.2)
3rd (12 days or more)	59 (31.2)	57 (96.6)	33 (55.9)	24 (40.7)	46 (85.2)	37 (80.4)	21 (56.7)	6 (16.2)
p-value **	< 0.01		< 0.01		< 0.01		< 0.01	
ICU admission required								
Yes	57 (30.0)	53 (93.0)	30 (52.6)	23 (40.3)	45 (28.1)	34 (75.6)	29 (64.4)	5 (11.1)
No	133 (70.0)	115 (86.5)	69 (51.8)	46 (34.6)	115 (71.9)	76 (66.1)	68 (59.1)	8 (6.9)
p-value **	0.20		0.40		0.24		0.41	
Time in ICU (days) (n = 57)								
Mean (SD)	13.8 (16.9)	14.5(17.3)	12.1(16.2)	17.5(18.6)	12.5 (16.1)	13.6 (16.8)	13.1 (15.7)	17.1 (15.1)
p-value **	0.26		0.28		0.29		0.20	
Time in ICU (tertiles)								
1st (2-14 days)	24 (42.1)	22 (91.7)	14 (58.3)	8 (33.3)	17 (85.0)	12 (70.6)	11 (64.7)	1 (5.8)
2nd (16-50 days)	18 (31.6)	16 (88.9)	10 (55.5)	6 (33.3)	16 (72.7)	11 (68.7)	9 (56.2)	2 (12.5)
3rd (60 days or more)	15 (26.3)	15 (100.0)	6 (40.0)	9 (60.0)	12 (80.0)	11 (91.7)	9 (75.0)	2 (16.7)
p-value **	0.43		0.39		0.31		0.57	
Mechanical ventilation required ^##^								
Yes	11 (6.5)	11 (100.0)	5 (45.5)	6 (54.5)	8 (5.0)	6 (75.0)	4 (50.0)	2 (25.0)
No	159 (93.5)	139 (87.4)	81 (50.9)	58 (36.5)	152 (95.0)	37 (75.5)	36 (73.5)	1 (2.0)
p-value **	0.21		0.31		0.97		0.02	

SD: standard deviation.

* Presence of one or more symptoms related to COVID-19 six months after hospital discharge;

** Chi-square test or Fisher’s exact test.

*** Other comorbidities reported had low frequency: asthma/bronchitis (n = 5), kidney disease (n = 5), lung disease (n = 5), and mental disorder/depression (n = 3);

^#^ Classified according to body mass index (BMI) and self-reported weight and height;

^##^ Mechanical ventilation required (n = 20): medical records did not have this information.

The prevalence of post-COVID-19 syndrome was higher among patients with high blood pressure (98.2%) and diabetes (98.4%), when compared to individuals without these comorbidities (74.7% and 83.3%, respectively; p-value < 0.01) six months after discharge, and 81.6% among patients with high blood pressure and 77.1% among patients with diabetes when compared to individuals without these comorbidities (53.4% and 65.2%, respectively) 12 months after discharge, with a significant difference only for high blood pressure at the second prospective follow-up. No significant difference was observed between patients with obesity and heart disease. The prevalence of post-COVID-19 syndrome and the presence of three or more symptoms were higher among patients with a higher number of comorbidities six months after discharge (p-value < 0.01) (Table 2).

The average length of stay of patients was 12.7 days; it was longer among patients who had at least one symptom six months after discharge (13.6 days) when compared to those who had no symptoms (5.6 days). Similar results were observed after 12 months. The prevalence of post-COVID-19 syndrome and the presence of three or more symptoms were also higher 6 and 12 months after discharge among patients in the tertile with the longest length of stay. Of the patients evaluated, 30.0% required admission to an ICU, with an average of 13.8 days in the ICU, and 6.5% required invasive mechanical ventilation and presented post-COVID-19 syndrome, with no statistically significant difference in post-COVID-19 syndrome and number of symptoms in relation to these variables (Table 2).

More than half of the patients presented muscular symptoms (58.9%) and neuropsychiatric symptoms (55.3%) six months after hospital discharge. These percentages remained high also after 12 months: 44.4% for muscular symptoms and 30.6% for neuropsychiatric symptoms. Fatigue was the most common symptom 6 and 12 months after hospital discharge (55.3% and 40.6%, respectively), followed by memory or decision-making problems (36.8%; 20%), hair loss (26.8%; 11.2%), dyspnea (16.3%; 7.5%), anxiety (14.2%; 8.1%), joint pain (12.1%; 6.8%), and attention deficit/concentration problems (10%; 1.2%) at 6 and 12 months, respectively (Table 3). When compared to sex, hair loss was the only symptom presenting a significant difference (p-value < 0.01), with a higher prevalence among female patients.

**Table 3:** Symptoms reported by COVID-19 patients assessed 6 and 12 months after hospital discharge, according to sex. Cuiabá, Mato Grosso State, Brazil, 2022.

Symptoms	6 months				12 months			
	Total (n = 190)	Male	Female	p-value *	Total (n = 160)	Male	Female	p-value *
	n (%)	n (%)	n (%)		n (%)	n (%)	n (%)	
Class								
Muscular	112 (58.9)	45 (57.7)	67 (59.8)	0.76	71 (44.4)	28 (43.1)	43 (45.2)	0.63
Neuropsychiatric	105 (55.3)	41 (52.6)	64 (57.1)	0.53	49 (30.6)	19 (29.2)	30 (31.6)	0.81
Dermatological	51 (26.8)	7 (9.0)	44 (39.3)	< 0.01	18 (11.2)	2 (3.1)	16 (16.8)	< 0.01
Cardiovascular	46 (24.2)	21 (26.9)	25 (22.3)	0.46	17 (10.6)	7 (10.7)	10 (10.5)	0.54
Pulmonary	34 (17.9)	16 (20.5)	18 (16.1)	0.43	15 (9.4)	6 (9.2)	9 (9.5)	0.95
Specific symptoms								
Fatigue	105 (55.3)	44 (41.9)	61(58.1)	0.53	65 (40.6)	24 (36.9)	41 (43.1)	0.62
Memory or decision-making problems	70 (36.8)	28 (40.0)	42 (60.0)	0.82	32 (20.0)	13 (20.0)	19 (20.0)	< 0.01
Hair loss	51 (26.8)	10 (19.6)	41 (80.4)	< 0.01	18 (11.2)	2 (3.1)	16 (16.8)	< 0.01
Dyspnea	31 (16.3)	13 (41.9)	18 (58.1)	0.61	12 (7.5)	4 (6.1)	8 (8.4)	0.59
Anxiety	27 (14.2)	8 (29.6)	19 (70.4)	0.19	13 (8.1)	4 (6.1)	9 (9.5)	0.45
Joint pain	23 (12.1)	13 (56.5)	10 (43.5)	0.24	11 (6.8)	5 (7.7)	6 (6.3)	0.73
Attention deficit/Concentration problems	19 (10.0)	8 (42.1)	11 (57.9)	0.92	2 (1.2)	2 (3.1)	-	0.08
Palpitation	18 (9.5)	8 (44.4)	10 (55.6)	0.75	7 (4.4)	4 (6.1)	3 (3.1)	0.36
Asthenia/Muscle weakness	14 (7.4)	2 (14.3)	12 (85.7)	0.03	6 (3.7)	1 (1.5)	5 (5.2)	0.22
Dizziness	10 (5.3)	3 (30.0)	7 (70.0)	0.46	5 (3.1)	1 (1.5)	4 (4.2)	0.34
Persistent headache	7 (3.7)	1 (14.3)	6 (85.7)	0.49	3 (1.9)	-	3 (3.1)	0.14
Cough	5 (2.6)	4 (80.0)	1 (20.0)	0.38	3 (1.8)	2 (3.1)	1 (1.1)	0.35
Depression	4 (2.1)	2 (50.0)	2 (50.0)	0.71	2 (1.2)	-	2 (2.1)	0.23
Taste disorder	5 (2.6)	2 (40.0)	3 (60.0)	0.96	-	-	-	-
Sleep disorder	2 (1.0)	-	2 (100.0)	0.23	2 (1.2)	1 (1.5)	1 (1.1)	0.78
Chest pain	2 (1.0)	-	2 (100.0)	0.23	1 (0.6)	-	1 (1.1)	0.40
Low mood	1 (0.5)	-	1 (100.0)	0.40	1 (0.6)	-	1 (1.1)	0.40
Sweating	1 (0.5)	1 (100.0)	-	0.23	-	-	-	-
Dysphoria/Indisposition	-	-	-	-	3 (1.9)	-	-	-

* Chi-square test or Fisher’s exact test.

A higher prevalence of post-COVID-19 syndrome was observed six months after discharge among patients who reported a higher number of symptoms in the acute phase of COVID-19 (p-value < 0.01). When assessing each symptom specifically, a higher prevalence of post-COVID-19 syndrome was observed among patients who reported cough (92.2%), fever (92.4%), and body aches (95.9%), when compared to patients without these symptoms (77.5%, 79.3%, and 80.6%, respectively; p-value < 0.05). A higher number of persistent symptoms after hospital discharge was also observed among patients with these symptoms at the 6-month assessment (Table 4).

**Table 4:** Post-COVID-19 syndrome * and number of symptoms among patients assessed six months after hospital discharge, according to symptoms in the acute phase of COVID-19. Cuiabá, Mato Grosso State, Brazil, 2022.

Symptoms in acute phase	Total	Post-COVID-19 syndrome		p-value **	Symptoms			p-value **
		No	Yes		None	1 or 2	3 or more	
	n (%)	n (%)	n (%)		n (%)	n (%)	n (%)	
General	190 (100.0)	22 (11.6)	168 (88.4)		22 (11.6)	99 (52.1)	69 (36.3)	
Cough				< 0.01				0.02
No	49 (25.8)	11 (22.4)	38 (77.5)		11 (22.4)	22 (45)	16 (32.6)	
Yes	141 (74.2)	11 (7.8)	130 (92.2)		11 (7.8)	77 (54.6)	53 (37.6)	
Fever				< 0.01				< 0.01
No	58 (30.5)	12 (20.7)	46 (79.3)		12 (20.7)	31 (53.4)	15 (25.9)	
Yes	132 (69.5)	10 (7.6)	122 (92.4)		10 (7.6)	68 (51.5)	54 (40.9)	
Dyspnea				0.13				0.31
No	84 (44.2)	13 (15.5)	71 (84.5)		13 (15.5)	43 (51.2)	28 (33.3)	
Yes	106 (55.8)	9 (8.5)	97 (91.5)		9 (8.5)	56 (52.8)	41 (38.7)	
Body ache				< 0.01				< 0.01
No	93 (48.9)	18 (19.4)	75 (80.6)		18 (19.3)	48 (51.6)	27 (29.1)	
Yes	97 (51.1)	4 (4.1)	93 (95.9)		4 (4.1)	51 (52.6)	42 (43.3)	
Fatigue				0.13				0.13
No	101 (53.2)	15 (14.9)	86 (85.1)		15 (14.9)	55 (54.5)	31 (30.7)	
Yes	89 (46.8)	7 (7.9)	82 (92.1)		7 (7.9)	44 (49.4)	38 (42.7)	
Headache				0.26				0.52
No	136 (71.6)	18 (13.2)	118 (86.8)		18 (13.2)	70 (51.5)	48 (35.3)	
Yes	54 (28.4)	4 (7.4)	50 (92.6)		4 (7.4)	29 (53.7)	21 (38.9)	
Taste disorder				0.72				0.90
No	150 (78.9)	18 (12.0)	132 (88.0)		18 (12.0)	77 (51.3)	55 (36.7)	
Yes	40 (21.1)	4 (10.0)	36 (90.0)		4 (10.0)	22 (55.0)	14 (35.0)	
Sore throat				0.17				0.22
No	163 (85.8)	21 (12.9)	142 (87.1)		21 (12.9)	86 (52.8)	56 (34.3)	
Yes	27 (14.2)	1 (3.7)	26 (96.3)		1 (3.7)	13 (48.1)	13 (48.2)	
Runny nose				0.93				
No	163 (85.8)	19 (11.7)	144 (88.3)		19 (11.7)	84 (51.5)	60 (36.8)	0.92
Yes	27 (14.2)	3 (11.1)	24 (88.9)		3 (11.1)	15 (55.6)	9 (33.3)	
Smell disorder				0.46				0.73
No	165 (86.8)	18 (10.9)	147 (89.1)		18 (10.9)	86 (52.1)	61 (37.0)	
Yes	25 (13.2)	4 (16.0)	21 (84.0)		4 (16.0)	13 (52.0)	8 (32.0)	

* Presence of one or more symptoms related to COVID-19 six months after hospital discharge;

** Chi-square test or Fisher’s exact test.

When evaluated 12 months after hospital discharge, the proportion of post-COVID-19 syndrome was higher among patients who reported more than two symptoms in the acute phase of COVID-19 (68.7%). When comparing each symptom specifically, a higher prevalence of post-COVID-19 syndrome was observed among patients who reported cough (71.6%), fever (73.7%), and dyspnea (74.4%) when compared to patients without these symptoms (60%, 56.5%, and 61.4%, respectively; with no statistically significant difference) (Table 5).

**Table 5:** Post-COVID-19 syndrome * and number of symptoms among patients assessed 12 months after hospital discharge, according to symptoms in the acute phase of COVID-19. Cuiabá, Mato Grosso State, Brazil, 2022.

Symptoms in acute phase	Total	Post-COVID-19 syndrome		p-value **	Symptoms			p-value **
		No	Yes		None	1 or 2	3 or more	
	n (%)	n (%)	n (%)		n (%)	n (%)	n (%)	
General	160 (100.0)	50 (31.3)	110 (68.7)		50 (31.3)	97 (60.6)	13 (8.1)	
Cough				0.02				0.04
No	40 (25.0)	16 (40.0)	24 (60.0)		16 (40.0)	22 (55.0)	2 (5.0)	
Yes	120 (75.0)	34 (28.3)	86 (71.6)		34 (28.3)	75 (62.5)	11 (9.2)	
Fever				0.03				0.04
No	46 (28.7)	20 (43.5)	26 (56.5)		20 (43.5)	25 (54.3)	1 (2.2)	
Yes	114 (71.2)	30 (26.3)	84 (73.7)		30 (26.3)	72 (63.2)	12 (10.5)	
Dyspnea				0.07				0.18
No	70 (43.7)	27 (38.6)	43 (61.4)		27 (38.6)	37 (52.8)	6 (8.6)	
Yes	90 (56.2)	23 (25.6)	67 (74.4)		23 (25.5)	60 (66.7)	7 (7.8)	
Body ache				0.56				0.60
No	81 (50.6)	27 (33.3)	54 (66.7)		27 (33.3)	49 (60.5)	5 (6.2)	
Yes	79 (49.4)	23 (29.1)	56 (70.9)		23 (29.1)	48 (60.7)	8 (10.1)	
Fatigue				0.02				0.03
No	87 (54.4)	34 (39.1)	53 (60.9)		34 (39.1)	45 (51.7)	8 (9.2)	
Yes	73 (45.6)	16 (21.9)	57 (78.1)		16 (21.9)	52 (71.2)	5 (6.8)	
Headache				0.52				0.66
No	113 (70.6)	37 (32.7)	76 (67.3)		37 (32.7)	68 (60.2)	8 (7.1)	
Yes	47 (29.4)	13 (27.6)	34 (72.4)		13 (27.6)	29 (61.7)	5 (10.6)	
Taste disorder				0.97				0.83
No	125 (78.1)	39 (31.2)	86 (68.8)		39 (31.2)	75 (60.0)	11 (8.8)	
Yes	35 (21.8)	11 (31.4)	24 (68.6)		11 (31.4)	22 (62.8)	2 (5.7)	
Sore throat				0.43				0.45
No	139 (86.8)	45 (32.4)	94 (67.6)		45 (32.4)	84 (60.4)	10 (7.2)	
Yes	21 (13.1)	5 (23.8)	16 (76.2)		5 (23.8)	13 (61.9)	3 (14.3)	
Runny nose				0.37				
No	137 (85.6)	41 (29.9)	96 (70.1)		41 (29.9)	85 (62.1)	11 (8.0)	0.65
Yes	23 (14.3)	9 (39.1)	14 (60.9)		9 (39.1)	12 (52.1)	2 (8.7)	
Smell disorder				0.46				0.68
No	139 (86.8)	42 (30.2)	97 (69.8)		42 (30.2)	85 (61.1)	12 (8.6)	
Yes	21 (13.1)	8 (38.1)	13 (61.9)		8 (38.1)	12 (27.1)	1 (4.8)	

* Presence of one or more symptoms related to COVID-19 six months after hospital discharge;

** Chi-square test or Fisher’s exact test.

## Discussion

The results of this study showed that 88.4% and 67.5% of patients who recovered from the acute phase of COVID-19 still had at least one symptom at the 6-month and 12-month assessments, respectively, and more than 30% had three or more symptoms of the disease at both interviews, with fatigue, dyspnea, joint pain, hair loss, and anxiety as the most common symptoms. The prevalence of post-COVID-19 syndrome was higher among older patients, with comorbidities, and lower per capita income. The results highlighted that post-COVID-19 syndrome affected more patients who presented higher severity in the acute phase, considering the length of stay and required admission to the ICU.

The prevalence of post-COVID-19 syndrome between 6 and 12 months after recovery from the acute phase ranges from 61% to 87% in international ^13^ and national literature ^20^, in agreement with the findings of our study. In Brazil, few studies have evaluated the presence of persistent symptoms of COVID-19 ^20^ ^,^ ^21^ ^,^ ^22^ ^,^ ^23^; this is the first study conducted in Mato Grosso, a state with a high cumulative number of deaths due to COVID-19, ranking second among the states of Brazil ^1^.

The frequency of symptoms in our study was higher than that reported in a prospective study conducted in Brazil with hospitalized patients; of these, 61% presented at least one symptom after six months, with fatigue and memory loss as the most frequent symptoms ^20^. This result can be attributed to the fact that in our study, 83.2% of respondents do not think they are recovered from COVID-19.

Although the 6-month assessment found no significant difference in the proportion of patients with post-COVID-19 syndrome according to sex, the 12-month assessment showed a higher number of female patients with three or more symptoms when compared to male patients. This result is similar to that found in a cohort in the Amazon Region that identified female patients, non-white skin color, and high BMI as independent predictors of a higher number of symptoms in post-COVID-19 syndrome ^22^, in agreement with other studies ^11^ ^,^ ^13^ ^,^ ^23^.

A higher prevalence of post-COVID-19 syndrome among individuals of older age, lower income, and without a job six weeks after discharge was consistent with the findings of studies conducted in other countries ^13^ and in Brazil assessing this theme ^20^. Also, the impact on physical and cognitive functions in individuals with post-COVID-19 syndrome can prevent them from returning to work, which consequently affects the family income ^20^. This difference in relation to the current job was not observed 12 weeks after discharge, which can be explained by the time from discharge to the 12-month interview, with consequent decrease in persistent symptoms among patients. Also, progress was seen with the economic growth in the country ^24^.

It should be noted that post-COVID-19 syndrome has disproportionately affected social groups; and social, economic, environmental, and political factors preceding the pandemic may have possibly contributed to health disparities ^25^. Then, inequality acts as a “threat enhancer”, interacting with the post-COVID-19 syndrome to increase the vulnerability of society as a whole ^25^.

Regarding comorbidities, prospective studies correlate preexisting comorbidities with post-COVID-19 syndrome, such as high blood pressure, cardiovascular disease, acute heart injury, and diabetes mellitus ^5^ ^,^ ^26^, consistent with the findings of our study. This relationship may be related to the fact that SARS-CoV-2 infection can cause an excessive release of cytokines (proinflammatory, profibrotic, and immune response regulator proteins), which results in aggravation of inflammatory mechanisms. Also, virus infection in the endothelial cell and consequent endothelial dysfunction is among the explanations for a more frequent occurrence of severe cases and post-COVID-19 syndrome among patients with comorbidities related to blood vessels ^27^. Also, patients hospitalized due to COVID-19 may experience worsening of preinfection comorbidities, including high blood pressure and diabetes mellitus ^5^.

As expected, the presence of symptoms after 6 and 12 months was higher among patients who were hospitalized for a longer period, which may be related to the greater severity of the disease in the acute phase. Studies found in the literature have reported a higher risk of long-term symptoms in patients requiring ICU admission and/or ventilatory support than in non-severe patients in terms of acute infection status ^28^ ^,^ ^29^. A recent study conducted in Brazil found a higher frequency of persistent symptoms among critical patients in the acute phase when compared to patients with mild and moderate levels ^21^.

Among the persistent symptoms among studied patients, muscular and neuropsychiatric symptoms were more frequent, with fatigue and dyspnea presenting similar results in other studies ^5^ ^,^ ^28^ ^,^ ^30^, and may be associated with recurrent visits to the health service ^31^. A possible explanation for these symptoms would be the persistence of residual fibrotic lung areas, resulting from an ineffective healing stage after the initial acute inflammatory response of the disease ^32^.

The COVID-19 pandemic has an impact on the mental health of infected and noninfected people ^33^. Survivors are at high risk of psychiatric outcomes, such as anxiety and depression ^34^, with the frequency of depressive symptoms more than 12 weeks after SARS-CoV-2 infection ranging from 11% to 28% ^35^. Our study found 55.3% of patients with neuropsychiatric conditions at the 6-month assessment and 30.6% at the 12-month assessment, with 14.2% of patients reporting anxiety and 2.1% depression at six months and 8,1% reporting anxiety and 1.2% depression at 12 months. Symptoms of depression can be at least partially explained by the exacerbation of inflammatory mechanisms mentioned above, given the evidence of association between inflammation and depression and that symptoms of depression in post-COVID-19 syndrome is proportional to the systemic inflammation measured in acute phase of the infection ^35^. Also, psychological symptoms may be caused by an indirect effect of virus infection, such as fear of dying from the disease, reduced social contact, loneliness, incomplete recovery of physical health, and job loss ^34^.

Memory problems are among the neuropsychiatric symptoms reported by the patients ^36^. These results indicate that individuals who have recovered from COVID-19 have a worse performance in cognitive tests in several domains than it would be expected. This impairment is proportional to the severity of symptoms and more commonly found among patients who were hospitalized ^36^. Also, memory problems after SARS-CoV-2 infection are possibly not due to exclusively neurological damage, but may be associated with psychiatric disorders and worsening of pre-existing cognitive issues. Further research is needed to provide a better understanding of all neurological disorders associated with COVID-19 and their cognitive manifestations ^36^ ^,^ ^37^.

Our findings report hair loss among the main symptoms identified 6 and 12 months after discharge, and among the dermatological symptoms of post-COVID-19 syndrome listed in the literature ^18^, possibly due to its pathogenesis, clinical course, and treatment ^32^ ^,^ ^38^. A study by Saceda-Corralo et al. ^39^ identified that hair loss may be a sequela in COVID-19 survivors, observed almost exclusively in women, in agreement with our findings that also identified a higher prevalence of hair loss hair among women when compared to men. The result of a study by Zheng et al. ^40^ indicated that women develop stronger humoral immune and cellular responses to COVID-19, which can extend symptom manifestations and cause sequelae such as hair loss. A higher prevalence of hair loss among women can also be explained by hormonal changes and stress that can be caused by the disease ^41^.

The results obtained in our study highlight a higher prevalence of post-COVID-19 syndrome among patients who had symptoms such as cough, fever, and body aches in the acute phase of the disease, which can be explained by the relationship between these symptoms and disease severity. These results agree with findings in the literature that report an association between disease severity in the acute phase and persistent symptoms, such as fatigue, dyspnea, muscle weakness, and stress ^28^ ^,^ ^42^.

Although fever is a common symptom in the acute phase, it usually improves over time and has not been observed among the main symptoms after recovery from the acute phase. A meta-analysis study on long-term symptoms in COVID-19 survivors found higher frequency of fatigue, dyspnea, cough, body aches, depression, anxiety, memory loss, and insomnia at one-year follow-up, but did not report fever among persistent symptoms ^13^ ^,^ ^43^.

In our study, less than half of the patients had completed the immunization schedule before admission, with no significant difference in the number of persistent symptoms according to the vaccine doses. However, the important role of vaccination to promote a significant reduction of the number of infection, reinfection, and serious cases must be considered ^44^. The fact that our study only analyzed patients who required hospitalization and did not evaluate the last dose date in relation to the hospitalization date may have prevented the analysis of this information, since studies have attempted to explain a lower probability of long-term symptoms among immunized individuals ^16^ ^,^ ^45^.

Among the limitations of our study, in addition to those mentioned above, convenience sampling should be highlighted, which prevents generalization of findings, and the failure to assess post-COVID-19 syndrome by differentiating virus variants. The definition of post-COVID-19 syndrome adopted should also be considered, based on the interviewee’s report of the presence of one or more symptoms from the list of 24 symptoms in the questionnaire applied 6 and 12 months after hospital discharge, according to other observational studies ^5^ ^,^ ^9^. Our study did not adopt the definition of the World Health Organization (WHO) issued in October 2021, which characterizes post-COVID-19 syndrome as the presence of symptoms three months from the onset of COVID-19 with symptoms that last for at least two months and cannot be explained by an alternative diagnosis. However, the WHO recognizes that 15.1% of individuals infected by SARS-CoV-2 present persistent symptoms 12 months after the onset of the infection, with the main symptoms including fatigue, dyspnea, and cognitive issues, such as that observed in our study ^46^. Also, with the variety of symptoms mentioned in the literature for post-COVID-19 syndrome, the list of 24 symptoms used in the questionnaire may have been a limitation, considering that more than 200 symptoms have been identified, affecting multiple organs ^46^. Also, a possible memory bias cannot be ignored for some retrospective questions and symptoms in the acute phase during hospital admission.

Future studies with representative sample and identification of virus lineage can contribute to knowledge about post-COVID-19 syndrome and how the COVID-19 pandemic evolves over time.

Our results suggest that a high number of patients may present post-COVID-19 syndrome within one year of hospitalization, being more frequent among patients of older age, with comorbidities, lower per capita family income, without a job, and among those with more serious COVID-19 in the acute phase. Our study can provide information about the prevalence of persistent symptoms, requiring a broad assessment of primary care about the long-term effects and impact on quality of life of symptoms and, if possible, support patients who may need treatment of high complexity.
